# Wnt pathway reprogramming during human embryonal carcinoma differentiation and potential for therapeutic targeting

**DOI:** 10.1186/1471-2407-9-383

**Published:** 2009-10-29

**Authors:** Grace E Snow, Allison C Kasper, Alexander M Busch, Elisabeth Schwarz, Katherine E Ewings, Thomas Bee, Michael J Spinella, Ethan Dmitrovsky, Sarah J Freemantle

**Affiliations:** 1 Department of Pharmacology and Toxicology, Dartmouth Medical School, Hanover, NH 03755, USA; 2 Department of Medicine, Dartmouth Medical School, Hanover, NH 03755, USA; 3 Norris Cotton Cancer Center, Dartmouth-Hitchcock Medical Center, Lebanon, NH, 03756, USA

## Abstract

**Background:**

Testicular germ cell tumors (TGCTs) are classified as seminonas or non-seminomas of which a major subset is embryonal carcinoma (EC) that can differentiate into diverse tissues. The pluripotent nature of human ECs resembles that of embryonic stem (ES) cells. Many Wnt signalling species are regulated during differentiation of TGCT-derived EC cells. This study comprehensively investigated expression profiles of Wnt signalling components regulated during induced differentiation of EC cells and explored the role of key components in maintaining pluripotency.

**Methods:**

Human embryonal carcinoma cells were stably infected with a lentiviral construct carrying a canonical Wnt responsive reporter to assess Wnt signalling activity following induced differentiation. Cells were differentiated with all-*trans *retinoic acid (RA) or by targeted repression of pluripotency factor, POU5F1. A Wnt pathway real-time-PCR array was used to evaluate changes in gene expression as cells differentiated. Highlighted Wnt pathway genes were then specifically repressed using siRNA or stable shRNA and transfected EC cells were assessed for proliferation, differentiation status and levels of core pluripotency genes.

**Results:**

Canonical Wnt signalling activity was low basally in undifferentiated EC cells, but substantially increased with induced differentiation. Wnt pathway gene expression levels were compared during induced differentiation and many components were altered including ligands (WNT2B), receptors (FZD5, FZD6, FZD10), secreted inhibitors (SFRP4, SFRP1), and other effectors of Wnt signalling (FRAT2, DAAM1, PITX2, Porcupine). Independent repression of FZD5, FZD7 and WNT5A using transient as well as stable methods of RNA interference (RNAi) inhibited cell growth of pluripotent NT2/D1 human EC cells, but did not appreciably induce differentiation or repress key pluripotency genes. Silencing of FZD7 gave the greatest growth suppression in all human EC cell lines tested including NT2/D1, NT2/D1-R1, Tera-1 and 833K cells.

**Conclusion:**

During induced differentiation of human EC cells, the Wnt signalling pathway is reprogrammed and canonical Wnt signalling induced. Specific species regulating non-canonical Wnt signalling conferred growth inhibition when targeted for repression in these EC cells. Notably, FZD7 repression significantly inhibited growth of human EC cells and is a promising therapeutic target for TGCTs.

## Background

Embryonal carcinoma (EC) cells are the undifferentiated and pluripotent component of germ cell nonseminoma tumors. Some EC cell lines can be induced to differentiate in response to cellular or pharmacological morphogens. These cells share many features in common with embryonic stem (ES) cells and their induced differentiation mimics critical stages of early embryogenesis [1]. Additional evidence indicating that EC and ES cells are closely related comes from their shared gene expression profiles, which are highly specific to germ cells and pluripotent ES cells [2]. These species include the transcription factors POU5F1 and Nanog, bone morphogenetic protein family member GDF-3, developmental pluripotency-associated gene 3 (DPPA3) and fibroblast growth factor 4 (FGF4).

The Wnt signalling pathway is essential for normal eukaryotic development and inappropriate activation of Wnt signalling occurs in many cancers [3]. Wnt ligands engage signal transduction through multiple receptors including the Frizzled transmembrane receptor family, co-receptors LRP5 and LRP6 and receptor tyrosine kinases, Ryk and ROR2 [4]. There are 19 Wnt ligand and 10 Frizzled receptor genes in the mammalian genome. The canonical Wnt-Frizzled signalling pathway results in stabilization of β-catenin allowing it to enter the nucleus and activate transcription of Wnt target genes by binding to T-cell factor/lymphoid enhancer factor (TCF/LEF) [5]. Frizzled receptors also play a key role in the planar cell polarity (PCP) pathway that is responsible for orienting cells relative to each other, and in a G protein-dependent pathway that triggers the release of calcium (Ca^2+^) [5]. The other Wnt receptors Ryk and Ror2 can signal through Src and JNK intermediates, respectively [6]. Wnt signalling proteins promote expansion of stem cells in diverse tissue contexts including the mammary gland, hematopoietic system, and the brain, underscoring the importance of this signalling pathway in stem cell maintenance [7].

The multipotent EC cell line NT2/D1 differentiates along a neuronal lineage in response to all-*trans *retinoic acid (RA) treatment, which is associated with loss of both self-renewal capacity and expression of pluripotent specific genes [8]. NT2/D1 cells were derived from a metastasis of a human testicular germ cell tumor (TGCT) and these retain the pathognomonic cytogenetic marker and cellular features of this malignancy [1,9]. In our initial studies to identify key species regulating early differentiation steps, several components of the Wnt signalling pathway were affected by RA-treatment [8]. This study sought to build on that prior work by comprehensively examining the expression and activity of Wnt species during induced differentiation of NT2/D1 cells and in a well characterized panel of TGCT cell lines including a derived RA-resistant cell line, NT2/D1-R1 [10]. Given that this pathway is important for both the maintenance of pluripotency and in regulating specific differentiation steps, it was hypothesized that during induced differentiation, the Wnt signalling machinery was reprogrammed in EC from a pathway supporting pluripotency to one promoting differentiation. Findings reported here provide substantial evidence confirming this hypothesis and these implicate the therapeutic potential of targeting the Wnt pathway in human EC and other TGCTs.

## Methods

### Cell Culture and Clonal Growth Assays

NT2/D1, NT2/D1-R1, Tera-1, 833K and 293T human cells were cultured in high glucose DMEM (InVitrogen, CA) containing 100 U/ml penicillin, 100 μg/ml streptomycin, 2 mM glutamine, and 10% fetal bovine serum (FBS) at 37°C under humidified 5% CO_2_. RA was dissolved in dimethyl sulfoxide (DMSO) as a 10 mM stock solution and stored in liquid nitrogen. Cells were treated with RA (10 μM), which was refreshed every 48 hours, while control cells were treated with DMSO vehicle alone. For colony formation assays, NT2/D1 and NT2/D1-R1 were independently plated at 5000 and 2500 cells per well, respectively, in six well tissue culture plates in triplicate. After 10-14 days, colonies were stained using Diff Quick (Imeb, Inc., CA) and quantified using the ColCount instrument (Oxford Optitronix, UK). Replicate experiments were performed to confirm results.

### siRNA Silencing

Double-stranded small interfering RNAs (siRNAs) with 19-nucleotide duplex RNA and a 2-nucleotide deoxythymidine overhang at the 3' region were synthesized (Dharmacon, CO and Qiagen, CA). Two different siRNAs were designed to target independently human POU5F1, FZD5 and FZD7 mRNAs. The sequences chosen were: FZD5-1 5'-ATCACGGTGCCCATGTGCC-3', FZD5-2 5'-TCCTAAGGTTGGCGTTGTA-3', FZD7-1 5'-AACGGCCTGATGTACTTTA-3', FZD7-2-2 5'-GGCCAGCTTGTGCCTAATA-3', POU5F1-2 5'-AGCAGCTTGGGCTCGAGAA-3' and POU5F1-3 5'-GAAAGAACTCGAGCAATTT-3'. Control siRNAs used were firefly luciferase pGL2 siRNA (5'-CGTACGCGGAATACTTCGA-3') and RISC free (5'-GCGCGCTTTGTAGGATTCG-3'), the latter is not a substrate for the RNAi silencing complex (RISC) (Dharmacon, CO). Transfection of siRNAs was accomplished with OligofectAMINE (Invitrogen, CA), as previously described [11]. To assess effects on cell growth, EC cells were trypsinized 24 hours after transfection and plated at 5 × 10^4 ^cells per well in 6-well plates. Triplicate wells were counted for each time point post-transfection and each experiment was performed at least three independent times. Results were expressed as mean ± standard deviation for a representative experiment. Subsequent assays for growth of small hairpin RNA (shRNA)-transduced cells were plated in the same way, but cell growth measurements were performed using the Cell Titre-Glo Assay (Promega, WI), which uses ATP content as an indicator of cell number.

### Immunoblot Analysis

Cells were lyzed in a modified radioimmune precipitation buffer, as described [12]. Protein concentrations were determined using the BCA protein assay kit (Thermo Scientific, IL). Proteins were separated by SDS-polyacrylamide gel electrophoresis (PAGE) and transferred to nitrocellulose membranes. Antibodies used and dilutions were FZD5, 1:500 (06-756 Upstate, NY), FZD7, 1:250 (AF198 R&D Systems, MN), active-β-catenin (ABC) 1:500 (05-665, Upstate, NY), β-catenin 1:2000 (MD, 610153, BD Transduction Laboratories), JNK and phospho-JNK 1:1000 (9258 and 9251, Cell Signaling Technology, Inc. MA), Oct3/4/POU5F1, 1:750 (H-134, Santa Cruz, CA), α-tubulin 1:1000 (CP06, Calbiochem, CA) and actin 1:2000 (C11, Santa Cruz, CA). Membranes were blocked in tris-buffered saline with 0.1% Tween 20 (TBS/Tween) plus 5% non-fat milk powder and primary antibodies were also diluted in 5% milk powder in TBS/Tween except for the ABC antibody which was diluted in 1% milk powder as previously described [13] and the JNK antibodies that were in 5% bovine serum albumin. Primary antibodies were detected with horseradish peroxidase conjugated secondary antibodies (Santa Cruz, CA and Amersham, IL) and visualized using the enhanced chemiluminescence system (Amersham, IL). For FZD5 and FZD7 detection, cellular lysates were not frozen and run under non-reducing conditions (without β-mercaptoethanol or boiling).

### Lentivirus Production and Luciferase Assays

The Topflash lentiviral vector was obtained from Dr. Karl Willert (University of California, San Diego). Silencing shRNA constructs were purchased (Open Biosystems, AL). To generate lentiviral particles, the desired plasmid and lentiviral packaging plasmids pCMV-dR8.2 dvpr and pCMV-VSVG (Addgene.com) were transfected into 293T cells using FuGENE 6 (Roche, Germany). Medium was changed 18 hours after transfection and viral particles were harvested twice over the next 72 hours. Cells were infected with the lentiviral supernatant for 24 hours in the presence of 4 μg/ml of polybrene (Sigma-Aldrich, MO). After 48 hours, cells were replated in 10-cm dishes. For shRNA lentiviral constructs, infected cells were selected by addition of 0.5 μg/ml puromycin (Sigma-Aldrich, MO). The Topflash lentiviral vector does not carry an antibiotic selectable marker and pools of cells were maintained for subsequent analyses. Luciferase activity was measured using the Promega (WI) luciferase assay system and normalized to protein concentrations, as above.

### Analysis of Wnt Signalling Pathway Genes

To assess regulated expression of Wnt pathway members following RA-mediated differentiation of NT2/D1 cells, real-time quantitative reverse transcription-polymerase chain reaction (RT-PCR) assays were performed. The RT2 Profiler™ PCR Array PAHS-043 (SABiosciences, MD) was used for these analyses. Total cellular RNA was isolated using TriReagent (Molecular Research Center, OH) and cDNA was synthesized from 1 μg of total RNA according to the manufacturer's instructions (SABioscience). Thermal cycling was performed using an Applied Biosystems 7500 Fast Real Time PCR System and real-time amplification data were obtained using ABI 7500 Fast System SDS software. Gene expression was normalized to internal controls (housekeeping genes) to determine fold changes in gene expression between test and control samples (SABioscience, MD). For the profiler arrays, the SYBR green PCR mix used was from -SABiosciences (MD). For real-time RT-PCR analyses of individual gene products, cDNA synthesis and SYBR green PCR reagents were purchased from Applied Biosystems (CA). Primer sequences used for real-time RT-PCR assays appear in additional file 1.

### Differentiation Marker Studies

Indirect fluorescence-activated cell sorter (FACS) analyses to evaluate RA-induced neuronal differentiation of NT2/D1 cells were performed using previously established methods [14]. Briefly, NT2/D1 cells were harvested by trypsinization and incubated with monoclonal antibodies to established human EC differentiation markers. The A2B5 antibody was isolated from a hybridoma culture and recognizes a neuronal epitope in RA-treated NT2/D1 cells (ATCC, VA). SSEA-3 (Developmental Studies Hybridoma Bank, University of Iowa, U.S.A.) recognizes an embryonal antigen, which is lost as EC cells differentiate [15]. Cells were indirectly assayed with Alexa 488 nM conjugated goat anti-mouse or goat anti-rat antibodies (Invitrogen, CA) and fluorescence was measured, as described [14].

### Statistics

Where a value for statistical significance is indicated a two sample two-tailed t-test assuming unequal sample variances was performed.

## Results

### Induction of canonical Wnt signalling during human EC differentiation

Stable integration of a canonical Wnt response element into NT2/D1 human EC cell pools allowed kinetic measurement of canonical Wnt activity. Transient transfection of a Wnt responsive reporter had been inconclusive in NT2/D1 cells induced to differentiate (data not shown). Similar studies examining the expression of the human cyclin D1 promoter in these cells also required stable integration of the promoter to recapitulate the expression pattern of the endogenous gene [16]. The chromatin of pluripotent cells is viewed as distinct from that of differentiated cells. It was reported that certain chromatin proteins in ES cells are hyperdynamic, binding loosely to chromatin [17]. This "breathing" chromatin both characterized pluripotent cells and was functionally relevant to their ability to differentiate into multiple cell types.

**Figure 1:**
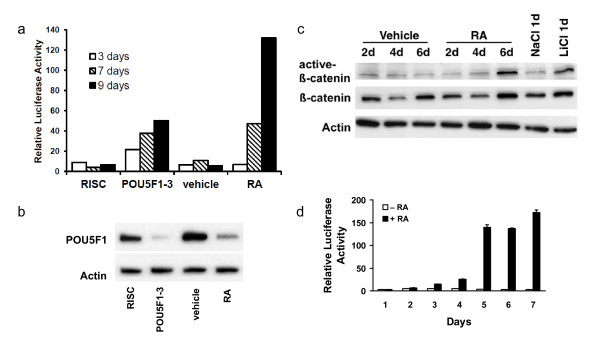
**Canonical Wnt signalling is induced with human EC differentiation**. NT2/D1 cells carrying a stably integrated multimerized canonical TCF-transcription factor binding site luciferase reporter were induced to differentiate. Luciferase activity was measured to determine the level of activity in the canonical Wnt signalling pathway and expressed relative to protein concentration. a) NT2/D1 cells transfected with siRNA for the transcription factor POU5F1 or control siRNA, and RA or vehicle control for 3, 7 and 9 days. b) Western validation of POU5F1 knockdown 3 days post siRNA transfection. POU5F1 levels of cells treated with RA were also measured after 3 days. c) Active-β-catenin levels in NT2/D1 cells following RA treatment. Lithium chloride (20 mM 24 hours) was used as a control to show activation of β-catenin, sodium chloride controlled for salt concentration. d) Time course of RA treatment over 7 days measuring integrated canonical Wnt reporter activity. Error bars represent standard deviation.

Little Wnt reporter luciferase activity was detected in basal cultures, but when RA was added, the levels of Wnt-responsive luciferase activity increased markedly over vehicle treated-matched control cells (Figures 1a, 1d). When these NT2/D1 cells were induced to differentiate by repression of the key pluripotency transcription factor POU5F1, the level of canonical Wnt signalling increased within 3 days and was over 6-fold elevated after 7 days (Figure 1a). Validation of POU5F1 knockdown by western analysis is shown in Figure 1b. POU5F1 levels were efficiently repressed by the siRNA and the repression of POU5F1 protein levels following 3 days of RA treatment is also shown. Under continued RA treatment, luciferase levels remained high, however beyond 9 days of POU5F1 siRNA treatment, luciferase levels fell as POU5F1 protein levels returned to normal (data not shown).

Protein lysates from treated cells were analysed using the activated β-catenin (ABC) antibody, which specifically recognizes the nonphosphorylated form of β-catenin [18]. The ABC epitope is revealed during canonical Wnt signalling and pharmacological inhibition of glycogen synthase kinase 3 (GSK3) by lithium chloride. The levels of ABC were clearly elevated following 6 days of RA treatment relative to vehicle treated cells (Figure 1c), and with 24 hours of lithium chloride treatment. The levels of total β-catenin were also elevated by these treatments, which reflects the stabilization of the hypophosphorylated form of β-catenin. The ABC levels were also elevated following knockdown of POU5F1, although to a lesser extent than with RA treatment, between 3 and 7 days post transfection (data not shown). Therefore, canonical Wnt signalling, as measured by Wnt reporter activity, is minimal in basally growing EC cells and activated as these cells are induced to differentiate.

### Multiple Wnt pathway genes are regulated during induced EC differentiation

**Figure 2:**
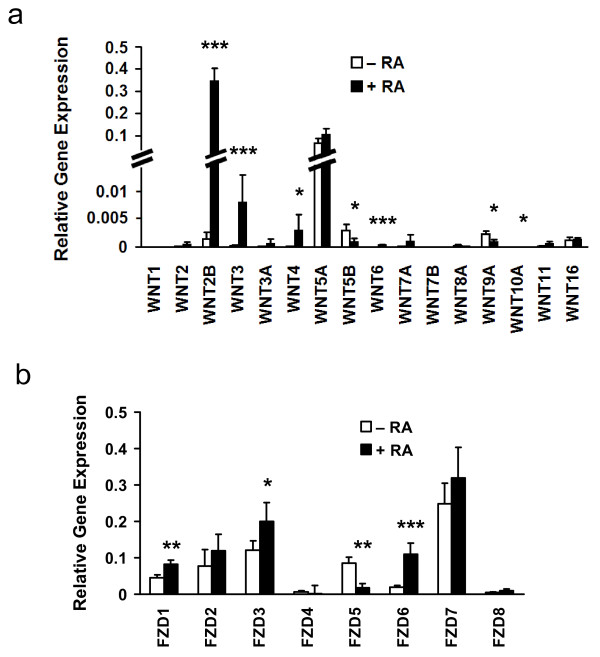
**Gene expression of Wnt ligands and Frizzled receptors in human EC cells**. Real-time RT-PCR arrays were used to determine the gene expression profiles of Wnt ligands and Fzd receptors in NT2/D1 cells relative to housekeeping genes. Graphs represent averages and standard deviations of at least 3 independent RNA samples. Graphs depict the gene expression profiles of a) Wnt ligands and b) Fzd receptors in NT2/D1 cells treated +/- RA for 4 days. (* *p *< 0.05, **p < 0.005).

To build on prior work, analyses of Wnt pathway gene expression were performed in NT2/D1 cells induced to differentiate and results were compared to controls. Differentiation was conferred with 4 days of RA (10 μM) treatment or with siRNA transfection to repress the pluripotency associated transcription factor POU5F1. RA treatment caused significant regulation of 6 of 16 Wnt ligands and 4 of 8 Frizzled receptors (Figure 2). Of the 84 genes analysed, 33 were significantly regulated following 4 days of RA treatment (Additional file 2). The analyses following siRNAs targeting POU5F1 revealed 11 of 84 species were significantly regulated, 10 of which were shared with the RA-regulated species (Additional file 3).

The levels of Wnt ligands in RA-treated and control cells are presented in Figure 2a. Of the 16 ligands examined, the most abundantly expressed in untreated cells was WNT5A. The cycle at which fluorescence exceeded a defined threshold (CT score) in this analysis varied widely for Wnt ligands with most being barely detected (i.e. CT score greater than 30). With RA-treatments, the levels of WNT2B increased markedly to levels above that of WNT5A. This is in agreement with our earlier studies using degenerate PCR primers for Wnt ligand analysis. In untreated cells, all the PCR products sequenced corresponded to WNT5A and after RA treatment the majority of PCR products sequenced were WNT2B with some WNT5A transcripts detected (data not shown). The prominent Wnt mRNAs expressed in this system are WNT5A and WNT2B. WNT3, WNT4, WNT6, and WNT10A were significantly up-regulated after 96 hours of RA-treatment and WNT5B and WNT9A were significantly down-regulated by this treatment (Figure 2a).

The expression of the Frizzled receptors varied less than for the Wnt ligands with moderate expression evident for each one (i.e., no CT score greater than 30). FZD1, FZD3 and FZD6 were each significantly up-regulated following RA-treatment and FZD5 was significantly down-regulated. FZD7 mRNA levels were the highest (i.e., it had the lowest CT score), but this was not as marked as for the differences detected in levels of the Wnt ligands.

The species most substantially up-regulated in response to both of these differentiation signals were WNT2B, SFRP4 and PITX2. In each case, these species were up-regulated over 100-fold after RA-treatment and 10- to 30-fold for cells transfected with POU5F1 siRNAs. WNT2B up-regulation had been observed during both RA- and hexamethylene bisacetamide (HMBA)-mediated differentiation of EC cells, confirming these changes are not confined to RA-treatment, but associated with induced differentiation [19]. PITX2 is a transcription factor associated with left-right asymmetry and is up-regulated by both RA and canonical Wnt signalling pathways [20-23]. SFRP4 is one of the secreted frizzled-related protein family members thought to compete with membrane bound frizzled receptors for Wnt binding [24].

**Figure 3:**
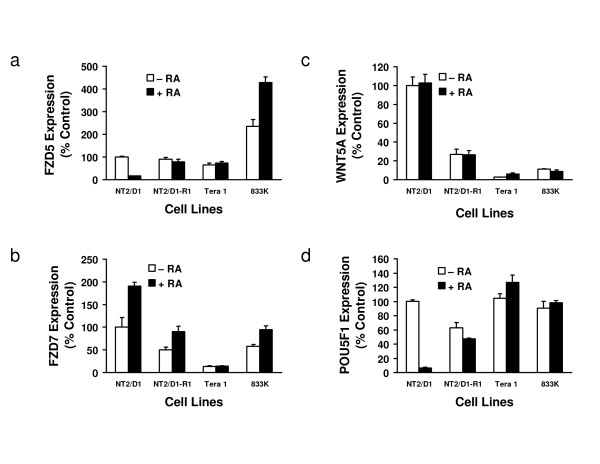
**Expression of FZD5, FZD7, WNT5A, and POU5F1 in human germ cell tumor cell lines**. Real-time RT-PCR assays were used to independently determine the expression of a) FZD5, b) FZD7, c) WNT5A, and d) POU5F1 in NT2/D1, NT2/D1-R1, Tera-1, and 833K cells. Gene expression profiles were determined in cells treated with +/- RA for 4 days. Values are expressed as percent of NT2/D1 (-RA), which served as a control. Graphs depict representative PCR assays performed in triplicate. Error bars represent standard deviation.

It was hypothesised that loss of the POU5F1 transcription factor in EC cells would result in repression of POU5F1 target genes and this was found for the known POU5F1-target gene, FGF4, which was repressed about 3-fold (Additional file 3). In RA-treated EC cells, FGF4 expression was repressed over 500-fold, suggesting mechanisms in addition to loss of POU5F1 act to reduce its mRNA levels. In these analyses PORCN, FRAT2, and FZD5 were each repressed following POU5F1 targeted repression, but this did not achieve statistical significance. Using independent primers for these species, each was verified as significantly repressed by both RA-treatment and POU5F1 repression in EC cells, implying these are potential POU5F1 target genes (Figure 3a and additional file 4).

### siRNA-mediated repression of FZD5 or FZD7 in EC cells

In searching for therapeutic targets of the Wnt signalling pathway, the G-protein coupled receptor (GPCR)-like Frizzled 5 (FZD5) and Frizzled 7 (FZD7) were chosen for further analyses. FZD5 was chosen because it is repressed as EC cells are induced to differentiate; FZD7 was selected since it was prominently expressed in EC and ES cells [2].

**Figure 4:**
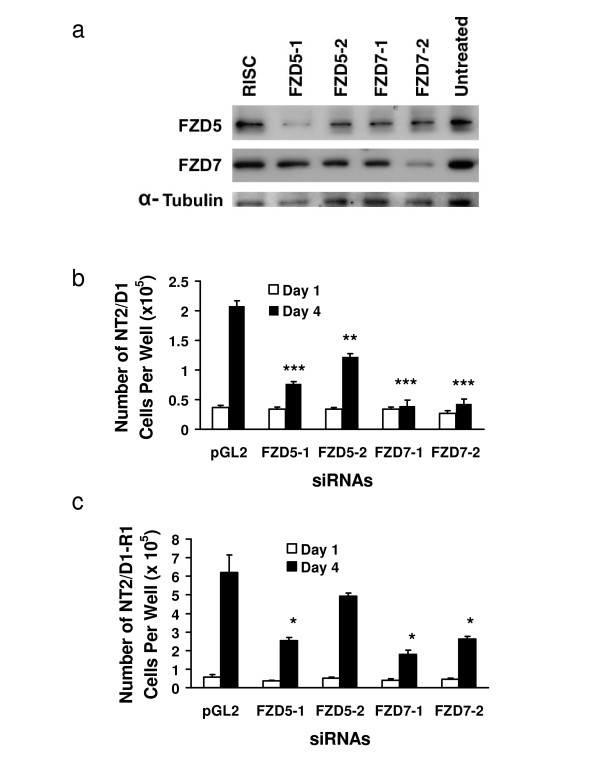
**Knockdown of FZD5 and FZD7 leads to a reduction in growth of EC cells**. NT2/D1 and NT2/D1-R1 cells were individually transfected with siRNAs to knockdown FZD5 or FZD7, or with a control siRNA construct, pGL2 or RISC. Graphs depict a representative experiment performed in triplicate. Error bars represent standard deviation. a) Expression of FZD5 and FZD7 proteins in NT2/D1 cells was quantified using immunoblotting on non-reducing gels. b) Number of NT2/D1 cells and c) number of NT2/D1-R1 cells after 4 days compared to initial number of seeded cells. (* *p *< 0.05, **p < 0.005, ***p < 0.0005).

Western analyses of NT2/D1 cell lysates two days after transfection, showed repression of FZD5 and FZD7 with siRNA sequences targeting each receptor (Figure 4a). Figure 4a is a representative immunoblot result and the cells lyzed for this were not from the same experiment as those used for the cell growth assay in Figure 4b. With siRNA FZD5-1 there is a clear repression of FZD5 protein while FZD5-2 has a reduced repression of FZD5 relative to the RISC and untreated cells. The levels of FZD5 protein were repressed in lysates from FZD7 siRNA transfected cells relative to the controls. This observation was made consistently with the two independent siRNA sequences targeting FZD7 and is viewed to be a consequence of FZD7 repression. The FZD7 protein levels were clearly repressed by the FZD7-2 siRNA, but not by the FZD7-1 siRNA or any of the other conditions. In a previous experiment harvested 3 days following siRNA transfection, this result was reversed and repression of FZD7 was only apparent with the FZD7-1 siRNA. This may be caused by a difference in the stability or effectiveness of the individual siRNA species or possibly a difference in the rate at which they silence their gene targets.

The day following siRNA transfection, cells were replated at equal densities and counted 24 and 96 hours later to determine growth effects. Both FZD5 and FZD7 repression resulted in significantly reduced cell numbers, with repression of FZD7 consistently achieving greater growth suppression than FZD5 in both NT2/D1 and its RA-resistant clone NT2/D1-R1 (Figure 4b and 4c). Experiments were performed to determine if siRNA mediated repression of FZD5 or FZD7 in EC cells induced differentiation, using established immunophenotypic markers recognized by A2B5 and SSEA-3, as in prior work [14]. These experiments did not reveal a substantial loss of the pluripotency-associated antigen SSEA-3 or gain of the neuronal specific antigen A2B5 (data not shown). Since the duration of siRNA-mediated repression might not be sufficient for the 6-day study time course, stable repression of FZD5 and FZD7 was sought using lentiviral shRNA infection of targeted cells.

One arm of the non-canonical Wnt signalling pathway activates JNK. To determine if either FZD5 or FZD7 were acting through JNK, the levels of activated-JNK in protein lystaes were examined from cells treated with siRNAs to FZD5 and FZD7. No significant changes in phospho-JNK levels were detected 3 days following siRNA transfection in any sample (additional file 5). In human synovial sarcoma cells this was sufficient time to see repression of JNK activation following siRNA-mediated repression of FZD10 [25]. Future studies aim to extend the time course examined following receptor repression and to overexpress the receptors to see if JNK activation increases.

### Stable repression of FZD5 and FZD7 in human EC cells

**Figure 5:**
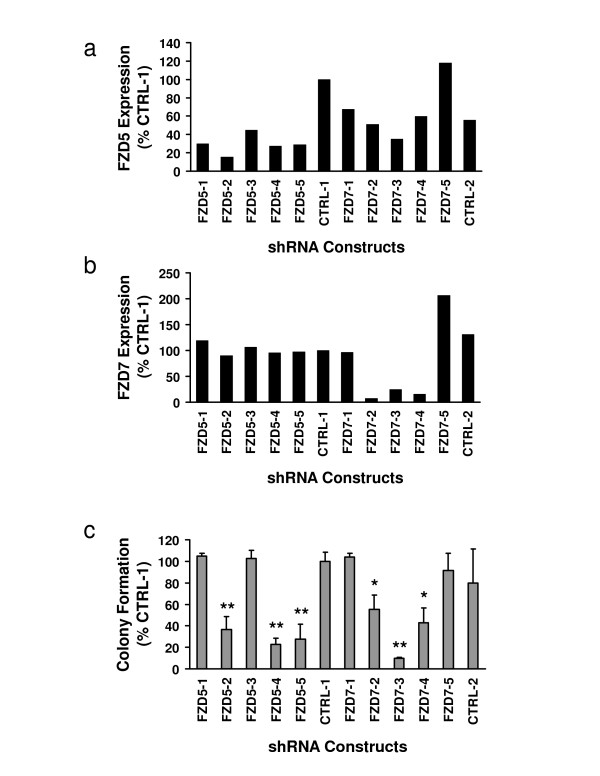
**Knockdown of FZD5 and FZD7 leads to a reduction in colony formation**. NT2/D1 cells were individually infected with lentiviral shRNAs for FZD5, FZD7, or a control shRNA. Graphs depict a representative experiment performed in triplicate. a) FZD5 and b) FZD7 expression profiles were determined using real-time RT-PCRs. Results presented are an average of duplicate readings. c) The ability of the cells to form colonies when plated at a low density is shown. Error bars represent standard deviation. (* *p *< 0.05, **p < 0.005).

Five separate lentiviral targeting constructs were individually tested for effective silencing of FZD5 and FZD7 (Figure 5a and 5b). Substantial repression of FZD5 mRNA was confirmed for all five constructs with most efficient repression detected with constructs FZD5-2, FZD5-4, and FZD5-5 (Figure 5a). Only 3 of the 5 FZD7 constructs appreciably repressed FZD7 mRNA and these were FZD7-2, FZD7-3 and FZD7-4. For FZD5, the constructs resulting in the most efficient repression of FZD5 mRNA levels also produced a significant inhibition of EC cell colony formation (Figure 5c). For FZD7, the 3 constructs showing efficient knockdown of FZD7 mRNA also showed significant repression of colony formation (Figure 5c). Construct FZD7-3 achieved the greatest growth inhibitory effect, but also exhibited off-target effects since it did not confer the highest repression of FZD7 expression and also caused moderate reduction of FZD5 mRNA. Cell growth experiments assessing cell number over 4 days confirmed the colony formation assay findings (data not shown).

**Figure 6:**
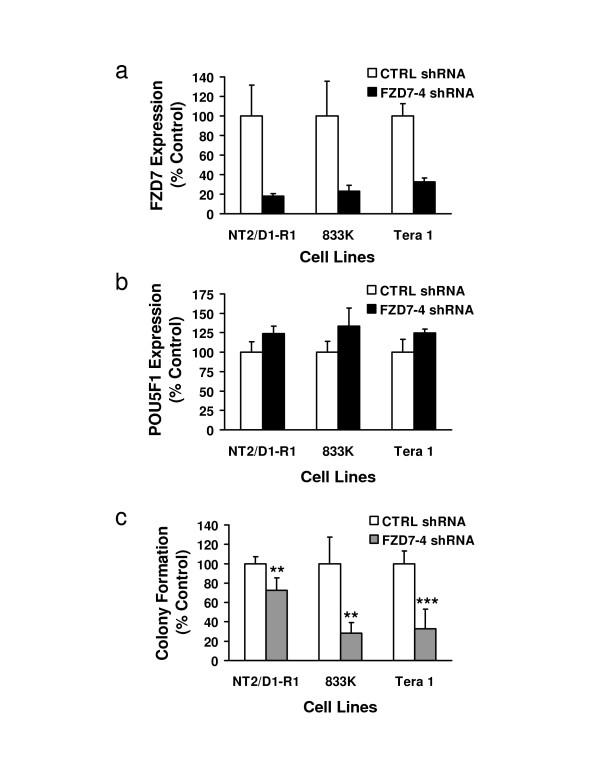
**Knockdown of FZD7 reduces colony formation, but does not cause loss of POU5F1 expression in EC cells**. NT2/D1-R1, 833K, and Tera-1 cells were each infected with lentiviral shRNAs to knockdown FZD7 expression. Graphs depict a representative experiment performed in triplicate, displayed as percent of control for each cell line. Error bars represent standard deviation. Real-time PCR assays were used to determine a) FZD7 gene expression and b) POU5F1 gene expression. c) Colony formation ability of FZD7 shRNA infected cells versus controls is displayed.

To determine if stable repression of FZD5 or FZD7 in EC cells induced differentiation, expression of immunophenotypic differentiation-specific extracellular markers recognized by A2B5 and SSEA-3 were each examined. Cells were selected on puromycin for at least 14 days before this drug was removed for 48 hours and cells were harvested for analyses. As with the siRNA-mediated repression of these receptors described above, there were no consistent changes in differentiation marker expression (data not shown). Expression patterns of several pluripotency-associated factors were examined including POU5F1, Nanog and FGF4. These species were not regulated by FZD5 and FZD7 repression (Figure 6b and data not shown). Thus, repression of FZD5 and FZD7 expression in human NT2/D1 EC cells inhibited growth, but did not impact expression of several key pluripotency factors.

### Inhibition of WNT5A expression in EC cells

WNT5A was the most highly expressed Wnt ligand uncovered in basally growing NT2/D1 cells. The shRNA lentiviral vectors to silence WNT5A expression were transduced to determine if loss of this ligand impacted EC cellular growth or differentiation. Efficient repression of WNT5A was achieved with three different lentiviral constructs and NT2/D1 colony formation was inhibited (additional file 6). The degree of knockdown of WNT5A mRNA did not correlate with colony formation inhibition. Construct WNT5A-5 had the most efficient knockdown while construct WNT5A-2 caused the greatest inhibition of colony formation. Ideally the protein levels of WNT5A would have been examined to confirm which shRNA construct gave the most efficient knockdown at the protein level. It is also possible that the WNT5A-2 construct caused off-target effects, which led to growth inhibition beyond that seen for WNT5A alone. Repression of WNT5A in NT2/D1 cells did not repress expression of pluripotency-associated factors POU5F1, FGF4 or Nanog (data not shown).

### Inhibition of FZD7 inhibits human EC cell growth

To ensure that the growth inhibitory effects seen in NT2/D1 cells were not cell line specific, other human EC and germ cell tumor cell lines were infected with the lentiviral constructs targeting FZD5 and FZD7. Transduced cells were examined for changes in cell growth and pluripotency-associated gene expression. The cell lines used were NT2/D1-R1 an RA resistant clone of NT2/D1 cells [10], Tera-1 and 833K cells, which were derived from lung and abdominal testicular cancer metastases, respectively [26,27]. Expression levels of FZD5, FZD7, WNT5A and POU5F1 were first examined to confirm each line was expressing similar levels of these species (Figure 3). Cells were analyzed +/- RA-treatment (10 μM) and, as expected, only NT2/D1 cells appreciably responded to RA-treatment by repressing FZD5 and POU5F1 expression. NT2/D1-R1 cells were selected for RA resistance and Tera-1 and 833K cells were studied since they were known not to differentiate with RA-treatment [28]. Untreated NT2/D1, NT2/D1-R1 and Tera-1 cells expressed similar amounts of FZD5 mRNA relative to the GAPDH expression levels and 833K cells expressed 2-4-fold higher levels. Tera-1 cells had approximately 10-fold lower and 833K and NT2/D1-R1 cells had 2-fold lower levels of FZD7 mRNA expression levels relative to NT2/D1 cells. WNT5A mRNA levels were also about 10-fold lower in Tera-1 and 833K cells and 5-fold lower in NT2/D1-R1 than in NT2/D1 cells. POU5F1 mRNA levels were similar in all four untreated cell lines examined.

In colony formation and proliferation assays, FZD5 repression conferred using lentiviral vectors FZD5-2 and FZD5-4 did not produce consistent growth inhibitory effects in NT2/D1-R1, Tera-1 or 833K cells (data not shown). However, repression of FZD7 using constructs FZD7-2 and FZD7-4 consistently caused substantial growth inhibition in all the cells tested. A representative experiment is displayed (Figure 6). Expression levels of FZD7 measured by real-time-RT-PCR assays were reduced to between 15-30% of control levels in NT2/D1-R1, Tera-1 and 833K cells (Figure 6a). This was sufficient to inhibit cell growth, but did not impact expression of the pluripotency-associated gene POU5F1, Nanog or FGF4 (Figure 6b and data not shown). Therefore, while Frizzled receptor FZD7 is playing an important role in EC cell growth, it is not impacting key pluripotency factors in these cells.

## Discussion

Canonical Wnt signalling is associated with proliferation and is deregulated in cancer [3]. Wnt target genes include cyclin D1 and c-myc, both of which are also associated with proliferation and are over-expressed in several cancers [3]. Human NT2/D1 EC cells have high levels of cyclin D1 and c-myc, which are both repressed as these cells are induced to differentiate [16,29]. Based on this, it was hypothesised that canonical Wnt signalling would be active in the proliferative and pluripotent EC cells and repressed in these cells induced to differentiate with a concurrent loss of tumorigenicity. In fact, the opposite was observed with canonical Wnt signalling at low levels in basally growing cells and elevated levels as cells were induced to differentiate, exemplifying the paradoxical nature of Wnt signalling in ES and EC cells. This is also reflected in prior work where some studies report that canonical Wnt signalling supports ES cell self-renewal and pluripotency and others have found that canonical Wnt signalling can induce ES cell differentiation, as reviewed [30]. Different cell lines and methodologies were used in these reports and might account for the apparent discrepant results. Also, the level of nuclear β-catenin and the origin of ES cells might determine how these cells respond to canonical Wnt signalling activation.

The EC cells examined here respond in a similar way to murine embryos in which Wnt signalling is not evident until day 6.5 when reporter activity is seen at the posterior of the embryo where the primitive streak begins to form [31]. Embryoid bodies generated from Wnt reporter carrying ES cells also showed polarized Wnt activation, and the Wnt responsive cells were enriched for mesoendodermal gene expression [31,32]. Using a similar functional reporter assay in ES cells, it was found that transcriptional activity of the canonical Wnt pathway is minimal until the cells are induced to differentiate [33]. In immunohistochemical studies of pediatric TGCTs, the EC specimens examined had low-levels of nuclear β-catenin while teratomas, which are the differentiated counterpart of these tumors, had elevated levels of nuclear β-catenin [34,35]. In combination with the observations reported here, this confirms the similarities in Wnt signalling between EC and ES cells.

From these analyses, several Wnt receptors and ligands were abundantly expressed in undifferentiated EC cells and repression of several of these species inhibited EC cell growth. WNT5A was the most abundant Wnt mRNA of the 16 examined in this study and this ligand has been most commonly associated with non-canonical Wnt signalling [36]. It is likely that non-canonical Wnt pathways are functioning in human EC cells to maintain their cell division. The distinct chromatin structure of EC and ES cells may be responsible for the lack of β-catenin-mediated transcription and it has been proposed that one role of POU5F1 is to block canonical Wnt signalling [17]. It was hypothesised that inhibition of WNT5A, the dominant Wnt ligand in the NT2/D1 system, would result in a more marked phenotype than repression of single frizzled receptors. As this was not the case, this indicates that either other Wnt ligands are playing redundant roles in this system and/or that the frizzled receptors are functioning without ligand, as reviewed [4]. Additionally, while WNT5A mRNA levels were higher than that of other Wnt ligands, as assessed by real-time RT-PCR assays, it is not known if this relates to appreciably higher levels of Wnt protein or activity.

Many of the changes in Wnt gene expression seen in induced differentiation of EC cells are also seen in induced differentiation of ES cells, highlighting the use of the EC system as a model for Wnt signalling in ES cell biology. Other studies looking at POU5F1 target genes in human and mouse ES cells proposed that PORCN, FRAT2, and FZD5 were potential POU5F1 target genes [37,38]. In a bioinformatics study, POU5F1 transcription factor binding sites were conserved among mammalian FZD5 orthologs [39]. Both SFRP1 and SFRP2 are downregulated in response to RA-treatment of EC cells [8,40] and in response to POU5F1 repression in murine ES cells [37]. Of equal interest are the species strongly induced with both methods of differentiation, which include SFRP4, PITX2 and WNT2B. The induction of these species may be due to changes in chromatin resulting from the loss of POU5F1 activity or other mechanisms.

The dichotomous roles Wnt signalling plays in proliferation versus differentiation are especially evident in studies of neurogenesis. Differences in model systems and techniques may partially explain some of the discrepancies. However, it seems increasingly likely that the complexity of Wnt pathway allows for distinct signaling outputs within a cell. Recently, coactivator usage by β-catenin has been shown to determine proliferative versus differentiation gene transcription programs [41-43] and manipulation of specific coactivator levels could maintain pluripotency in murine ES and EC cells [42]. In addition to the increase in activated β-catenin seen as NT2/D1 cells are induced to differentiate, coactivator usage by β-catenin may also be changing and we aim to investigate this in future studies.

To target the Wnt signalling pathway, it was decided to specifically repress the expression of the FZD5 and FZD7 receptors. FZD5 was down-regulated in response to differentiation of NT2/D1 cells and in human melanoma cells FZD5 activation by WNT5A had been associated with elevated metastatic potential [44]. FZD5 repression was growth suppressive in NT2/D1 cells, but was not as effective in the other TGCT cell lines tested. FZD7 was highlighted in a study to identify species preferentially expressed in ES and EC cells [2] and was significantly growth suppressive in all the EC cell lines studied here. FZD7 has also been identified as a potential therapeutic target in hepatocellular and colorectal cancers [45,46].

Targeted repression of FZD7 was growth suppressive for all EC cells tested, but this did not cause differentiation of these cells. This is in contrast to a recent paper where shRNA-mediated repression of FZD7 decreased POU5F1 expression substantially in human ES cells and moderately in the human EC cell line NCCIT [47]. These differences may be due to the distinct features of the cell lines used. Also, human ES cells may respond differently than human EC cells. A single shRNA construct was used in the human ES cell study, which caused a 60-fold knockdown of FZD7 mRNA, whereas the greatest knockdown achieved with our panel of shRNA constructs was 20-fold repression. In agreement between these reports were the observed phenotypic cell changes. For the human ES cells, this was particularly evident at the edges of colonies, whereas the NT2/D1 cells became elongated and spindle-like with repressed FZD7 expression (data not shown). Many of the non-canonical Wnt signalling pathways target the cytoskeleton, as in melanoma where non-canonical signalling by WNT5a is found to correlate with metastatic potential and invasiveness [44]. Recently, it was reported that WNT5a polarizes the cytoskeleton, allowing chemokine directed motility in melanoma cell lines [48]. TGCTs are highly metastatic and may be utilizing this pathway to enter the blood system and then spread to other sites. Subsequent studies aim to examine the role of Wnt pathways in EC cell motility and differentiation-induced cytoskeletal changes. Initial focus will be on DAAM1 and profilin-1, two proteins involved in actin organization and non-canonical Wnt signalling and up-regulated as EC cells are induced to differentiate along a neuronal lineage (Additional file 2 and [49]).

The complexity of the Wnt signalling pathway is due to in part to the large number of potential ligands and receptors. In addition, challenges exist in developing pharmacological tools to specifically inhibit Wnt ligand activity that have made this pathway difficult to dissect. Wnt ligands were historically divided into those resulting in either canonical or non-canonical signalling. With the discovery of more Wnt receptors, non-Wnt ligands and Wnt-independent Frizzled activation, a new view of the Wnt pathway is being proposed: that it is the receptor and downstream effector context, in addition to ligand identity, which determines ultimate signalling output [4]. The established role of Wnt signalling in development and its misregulation in cancer and other diseases highlights the need for a more complete understanding of how this pathway works. Novel pharmacological tools to inhibit Wnt signalling are becoming available [50]. This will accelerate the evaluation of the therapeutic possibilities of Wnt pathway manipulation in human EC and other tumors.

## Conclusion

EC cells are the malignant counterparts of ES cells, with induced differentiation of EC cells mimicking many stages of early embryogenesis. Multiple components of the Wnt pathway are regulated as EC cells are induced to differentiate; many of these are also regulated in differentiating human ES cells. The human EC cell system is therefore an attractive model to explore Wnt signalling before confirming observations in the more practically complex ES cell context. Despite minimal canonical Wnt activity in basally growing EC cells, specific repression of Wnt receptor FZD7 resulted in consistent growth inhibition of human EC cells, implicating non-canonical Wnt pathways in maintaining EC cell proliferation. FZD7 is preferentially expressed in EC cells and is therefore an attractive therapeutic target for human ECs.

## Competing interests

The authors declare that they have no competing interests.

## Authors' contributions

GES, ACK, AMB, KEE, ES, TB and SJF carried out molecular and cell biology experiments, and participated in the design of the study. GES and AMB prepared the figures and aided in interpretation of the data and in manuscript preparation. MJS provided the initial observations and helped in the design of experiments and in interpretation of results. ED contributed to the overall scientific direction, experimental design and interpretation, and in manuscript preparation. SJF directed the study and prepared the manuscript. All authors read and approved the final manuscript.

## Pre-publication history

The pre-publication history for this paper can be accessed here:

http://www.biomedcentral.com/1471-2407/9/383/prepub

## Supplementary Material

Additional file 1 **Primers used in these analyses**. Primer sequences used for human genes analyzed. Click here for file

Additional file 2 **Gene expression changes in NT2/D1 cells treated with RA**. Raw Wnt array data from NT2/D1 cells treated +/- 10 μM RA for 96 hours. Click here for file

Additional file 3 **Gene expression changes in NT2/D1 cells treated with siRNA to POU5F1**. Raw Wnt array data from NT2/D1 cells treated with siRNA to POU5F1 or a control siRNA for 96 hours. Click here for file

Additional file 4 **Effect of differentiation on PORCN, FRAT2, and FZD5 expression**. NT2/D1 cells were treated with RA or transfected with siRNA for POU5F1 to induce differentiation. A real-time PCR assay was used to determine expression of PORCN, FRAT2, and FZD5, respectively. Expression is displayed as percent of control. Graphs depict an average of at least two RNA samples with real-time RT-PCR assays performed in triplicate. Error bars represent standard deviation. a) PORCN expression. b) FRAT2 expression. c) FZD5 expression. (**p < 0.005, ***p < 0.0005). Click here for file

Additional file 5 **Knock-down of FZD5 and FZD7 expression with siRNA does not impact the levels of activated JNK**. NT2/D1 cells were transfected with siRNA and cell lysates harvested after 72 hours. Lysates were blotted with antibodies specific for the activated form of JNK (phospho-JNK), total JNK and β-actin. Click here for file

Additional file 6 **Knock-down of WNT5A leads to a reduction in colony formation**. NT2/D1 cells were infected with lentiviral shRNAs to knockdown WNT5A expression, and results were compared to a control shRNA. Graphs depict a representative experiment performed in triplicate. Error bars represent standard deviation. a) WNT5A expression was measured using a real-time PCR assay. b) The ability of the cells to form colonies when plated at a low density is displayed. (* *p *< 0.05). Click here for file
