# Supplementary material for: Wnt pathway reprogramming during human embryonal carcinoma differentiation and potential for therapeutic targeting
Source: BMC Cancer. 2009 Oct 29;9:383. doi: 10.1186/1471-2407-9-383 (PMC2777936; doi:10.1186/1471-2407-9-383)
Supplement: Additional file 1 — Primers used in these analyses. Primer sequences used for human genes analyzed. [file 1471-2407-9-383-S1.doc]

Gene Primer Sequence

FGF4 Forward: GCCAGCCGGTTCTTCGT

Reverse: TTGGGAAGGAGAATCTCCTTGA

FRAT2 Forward: AGGCCGTCGGAGAAAACTTAA

Reverse: GGCCCGGGAAATTCTCAT

FZD5 Forward: CGGTGTGCCAGGAAATCAC

Reverse: CGTGTCGTGGTTGAACTGGTT

FZD7 Forward: CCGTACCTGGGCTACCGCTT

Reverse: GCGGGCGAAGCGCCTCTCCT

GAPDH Forward: TGCACCACCAACTGCTTAGC

Reverse: GGCATGGACTGTGGTCATGAG

PITX2 Forward: CCGAGTCCGGGTTTGGTT

Reverse: AGCTCGGCCTGCTGGTT

PORCN Forward: TCCTACATGGCTTCAGTTTCCA

Reverse: GCGCTTCCGGAGGACAT

POU5F1 Forward: AAGCGATCAAGCAGCGACTAT

Reverse: GGAAAGGGACCGAGGAGTACA

SFRP4 Forward: GATGTTGACTGTAAACGCCTAAGC

Reverse: GAATAACATAGCTGTAGTTTTTGCTGAGA

WNT5A Forward: CATGAACCTGCACAACAACGA

Reverse: GGCACTTGCAGGCCACAT

**Additional file 1.** Primers used in real-time RT-PCR assays.
