# Supplementary figures and images for: Wnt pathway reprogramming during human embryonal carcinoma differentiation and potential for therapeutic targeting
Source: BMC Cancer. 2009 Oct 29;9:383. doi: 10.1186/1471-2407-9-383 (PMC2777936; doi:10.1186/1471-2407-9-383)

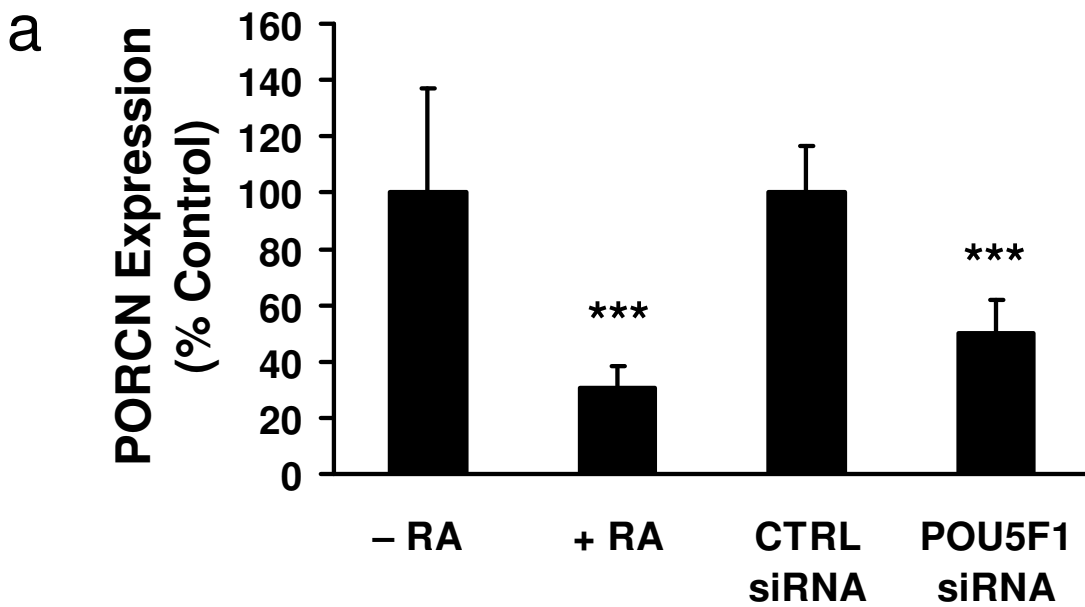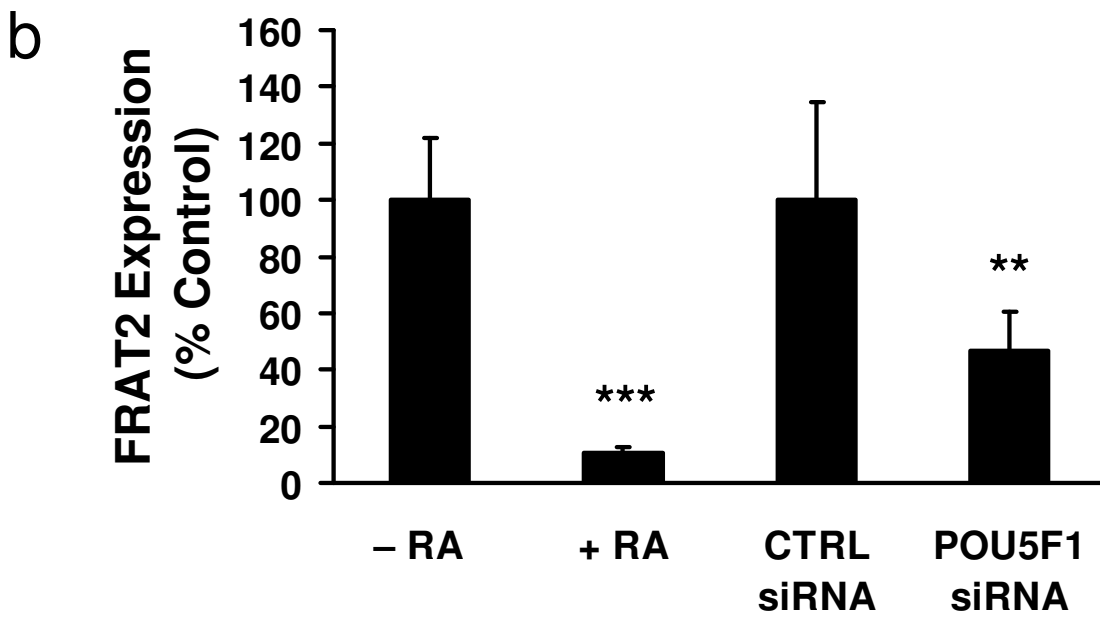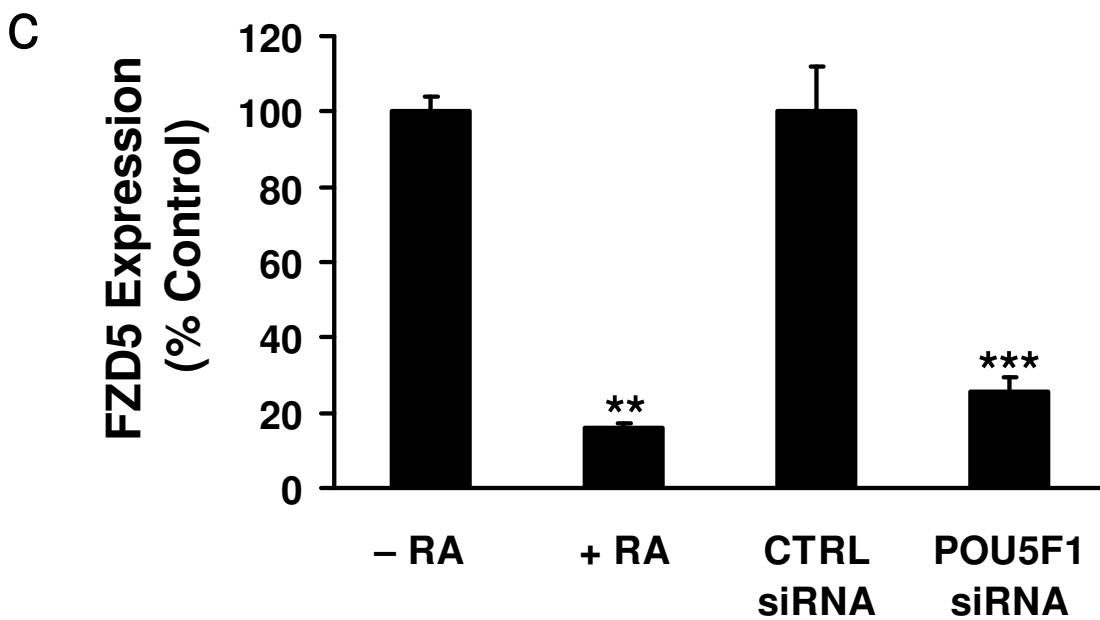

Supplement: Additional file 4 — Effect of differentiation on PORCN, FRAT2, and FZD5 expression. NT2/D1 cells were treated with RA or transfected with siRNA for POU5F1 to induce differentiation. A real-time PCR assay was used to determine expression of PORCN, FRAT2, and FZD5, respectively. Expression is displayed as percent of control. Graphs depict an average of at least two RNA samples with real-time RT-PCR assays performed in triplicate. Error bars represent standard deviation. a) PORCN expression. b) FRAT2 expression. c) FZD5 expression. (**p < 0.005, ***p < 0.0005). [file 1471-2407-9-383-S4.pdf]

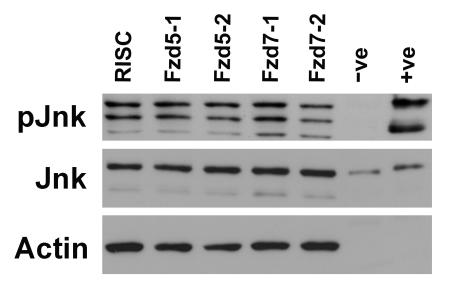

Supplement: Additional file 5 — Knock-down of FZD5 and FZD7 expression with siRNA does not impact the levels of activated JNK. NT2/D1 cells were transfected with siRNA and cell lysates harvested after 72 hours. Lysates were blotted with antibodies specific for the activated form of JNK (phospho-JNK), total JNK and β-actin. [file 1471-2407-9-383-S5.tiff]

a

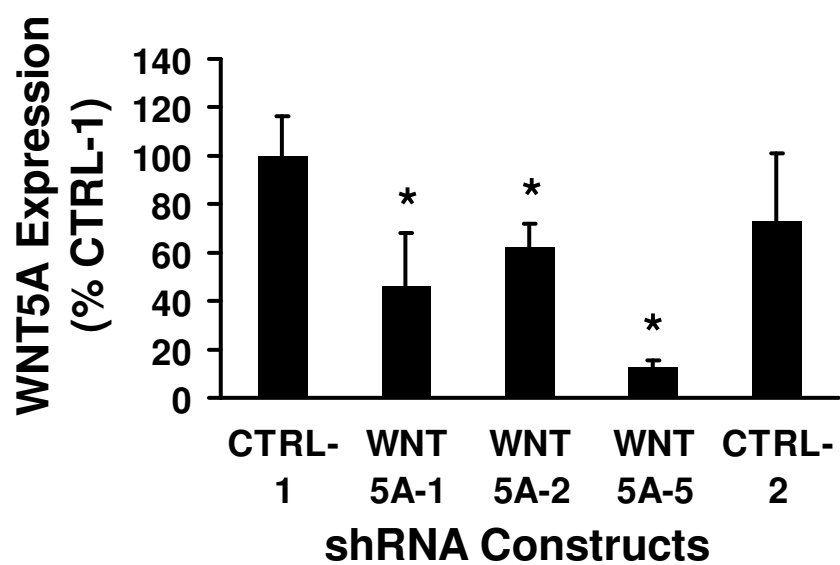

b

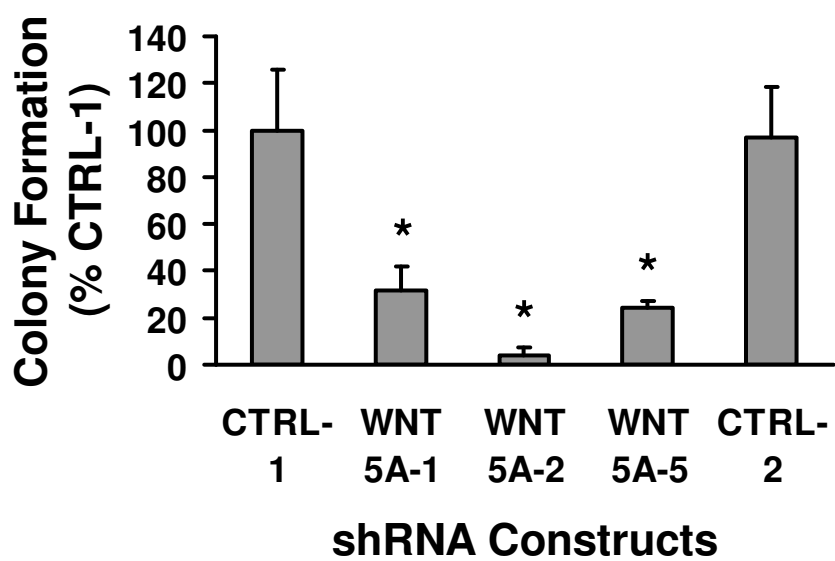

Supplement: Additional file 6 — Knock-down of WNT5A leads to a reduction in colony formation. NT2/D1 cells were infected with lentiviral shRNAs to knockdown WNT5A expression, and results were compared to a control shRNA. Graphs depict a representative experiment performed in triplicate. Error bars represent standard deviation. a) WNT5A expression was measured using a real-time PCR assay. b) The ability of the cells to form colonies when plated at a low density is displayed. (* p < 0.05). [file 1471-2407-9-383-S6.pdf]
